# A structural equation model of online learning: investigating self-efficacy, informal digital learning, self-regulated learning, and course satisfaction

**DOI:** 10.3389/fpsyg.2023.1276266

**Published:** 2024-01-11

**Authors:** Yi Zheng, Ao Xiao

**Affiliations:** ^1^School of Foreign Languages and Literatures, Chongqing Normal University, Chongqing, China; ^2^NYU Steinhardt Teachers of English to Speakers of Other Languages, New York University, New York, NY, United States

**Keywords:** online learning self-efficacy, online self-regulated learning, informal digital learning of English (IDLE), online course satisfaction, EFL learners, structural model

## Abstract

**Introduction:**

English as a Foreign Language (EFL) education increasingly relies on online learning, necessitating a nuanced understanding of crucial factors impacting learning experiences. This research investigates the intricate relationships among online learning self-efficacy, online self-regulated learning, informal digital learning of English (IDLE), and online course satisfaction within the unique context of EFL learners.

**Methods:**

The study involved 563 intermediate college students from various national universities in China. Structural Equation Modeling (SEM) was employed to analyze the data, providing comprehensive insights into the relationships among the identified variables.

**Results:**

The results revealed significant insights. Both online learning self-efficacy and IDLE exhibited direct and positive influences on online course satisfaction. Furthermore, the study uncovered that online self-regulated learning acted as a partial mediator in the connection between online learning self-efficacy and IDLE with online course satisfaction. This mediation implies that learners’ self-regulatory behaviors significantly affect how self-efficacy and informal digital language learning experiences impact overall satisfaction with online courses.

**Discussion:**

The findings highlight the pivotal role of nurturing learners’ self-efficacy beliefs, fostering IDLE, and promoting effective self-regulated learning strategies in the realm of online language learning. These initiatives are instrumental in enhancing learners’ satisfaction and success in online courses.

**Conclusion:**

The implications of these findings for EFL instruction are substantial. By emphasizing the importance of self-efficacy, IDLE, and self-regulated learning strategies, educators can significantly contribute to creating more satisfying and successful online learning experiences for EFL students.

## 1 Introduction

The 21st century has witnessed a swift transition toward digital information and media, significantly enhancing the role of information and communication technology (ICT) across diverse sectors, including education and language learning (Fernández-Gutiérrez et al., 2020; Rahimi and Fathi, 2022; Al-Rahmi et al., 2023; Bolaji and Jimoh, 2023). This surge in technology has become integral to educational frameworks, leading to a substantial rise in online courses and programs, consequently escalating enrollment rates (Arrosagaray et al., 2019; Abraham et al., 2022; Adilbayeva et al., 2022; Fathi et al., 2023).

Online courses play a pivotal role in augmenting learning experiences, particularly in the domain of English as a Foreign Language (EFL), known for providing engaging and authentic learning materials that significantly enhance EFL students’ academic performance (Liu et al., 2022; Asratie et al., 2023; Jiang et al., 2023; Yang and Xu, 2023).

Despite widespread integration of e-learning in second language acquisition (SLA), challenges persist regarding students’ affective and psychological states in online learning. While some learners find online education as effective as traditional classes (Sagarra and Zapata, 2008), others may not find the online environment satisfactory (Dizon and Thanyawatpokin, 2021). Learner satisfaction serves as a framework for evaluating and enhancing online learning, representing students’ perceptions of how well their learning requirements and goals are met (Hew et al., 2020). Acknowledged as a pivotal factor in determining the effectiveness of online courses, student satisfaction correlates with their performance and experience in virtual learning (Elshami et al., 2021; Abdelrady and Akram, 2022). Research indicates that students’ satisfaction in online courses significantly correlates with their academic success (Wei and Chou, 2020; Kim and Kim, 2021; She et al., 2021). Particularly in EFL contexts, online course satisfaction links to heightened engagement, determination, and reduced dropout rates, fostering motivation (Chen and Adesope, 2016; Fujii et al., 2022). Online courses differ from face-to-face settings, altering student-instructor and peer interactions (de Jong et al., 2020; Fathi and Ebadi, 2020; Wu et al., 2023). Neglecting students’ psychological needs in virtual environments may diminish motivation and confidence in pursuing language learning goals, potentially leading to decreased satisfaction and adverse academic outcomes (Abdullah, 2022; Ji et al., 2022).

In online language learning, several factors significantly influence learners’ experiences. Notably, learners’ self-efficacy, self-regulation, and engagement in informal digital learning of English (IDLE) play crucial roles (Lee, 2019; Lee and Sylvén, 2021). IDLE, a subset of computer-assisted language learning (CALL), attracts digital-native EFL learners and involves informal language learning through computer-based technologies like smartphones, computers, social media, and blogs (Lai, 2019; Soyoof et al., 2023). These self-directed, unstructured experiences offer authentic language learning opportunities (Liu et al., 2023). Research underscores the positive impact of IDLE on EFL students, enhancing language proficiency, cultural awareness, and communication skills (Lee, 2021; Lee et al., 2022; Liu et al., 2023). Engaging in IDLE activities contributes to improved language learning satisfaction by fostering proficiency and cultural understanding.

Despite growing interest in student satisfaction, little research has probed into its intricacies within L2 online courses, especially in China’s unique context (Gyamfi and Sukseemuang, 2018). This dearth of exploration emphasizes the urgency for further investigation in this critical area. Our study takes a pioneering step to uncover the pivotal roles of online learning self-efficacy, online self-regulated learning, and IDLE in predicting satisfaction among Chinese EFL students in the dynamic virtual classroom environment. It fills a void in the field by exploring these multifaceted elements, contributing substantially to the understanding of online language learning. Moreover, it specifically delves into the dynamics of Chinese EFL learners, an area scarcely examined before. Beyond theoretical advancement, this research carries significant practical implications, paving the way for targeted techniques and interventions to enhance learner satisfaction and success in the online language learning realm.

## 2 Literature review

### 2.1 Theoretical framework

This study is firmly anchored in the self-determination theory (SDT) proposed by Deci and Ryan (1985). SDT delves into the motivations and self-regulation of individuals across diverse contexts, with a specific emphasis on education. Central to SDT is the notion that individuals possess inherent psychological needs for autonomy, competence, and relatedness, which serve as prime drivers of motivation and exert a profound influence on their behaviors (Ryan and Deci, 2000).

*Autonomy* addresses the imperative for learners to experience a sense of choice and volition in their learning journey (Deci and Ryan, 2000). In the context of this study, it translates to learners feeling empowered to take charge of their online learning experiences, making informed choices about their learning activities, and setting their own educational objectives (Roca and Gagné, 2008). The *competence* facet of SDT revolves around learners’ intrinsic need to perceive themselves as effective and proficient in their learning pursuits (Deci and Ryan, 2015). Within the realm of online learning, learners with a robust sense of competence believe in their capacity to proficiently navigate digital platforms, actively engage in self-regulated learning practices, and ultimately accomplish their learning goals. Furthermore, *relatedness* underscores the importance of social connections and a sense of belonging within the learning environment (Deci and Ryan, 2000). In the online domain, this encompasses learners feeling a genuine connection to their peers, instructors, and the broader learning community.

In the context of online learning, learners’ *satisfaction* is intricately tied to the fulfillment of their fundamental psychological needs (Chen and Jang, 2010). When the online learning environment supports their autonomy (granting choice and control over their learning), competence (fostering perceived effectiveness and mastery), and relatedness (facilitating social interaction and connection), learners are more likely to experience satisfaction. Concerning *online self-regulated learning*, SDT provides valuable insights into the development of self-regulated learning skills within online environments (Hsu et al., 2019). Learners who perceive autonomy in setting their learning goals and strategies, competence in their ability to manage their learning, and relatedness with their peers and instructors are more likely to engage in effective self-regulated learning. In the domain of *online learning self-efficacy*, self-efficacy beliefs, a pivotal component of SDT, are closely intertwined with perceived competence (Chiu, 2022). Learners with a strong sense of self-efficacy in the online learning context are more inclined to believe in their capacity to succeed and are motivated to invest sustained effort and persistence in their learning endeavors. Applying SDT to IDLE, learners engaging in informal digital learning activities do so autonomously, driven by their choice to explore resources that pique their interest (Huang et al., 2019). This can significantly enhance their competence in the language and their sense of relatedness to the global online community of language learners.

By anchoring our study in SDT, we establish a unified theoretical framework that elucidates the intricate interplay between course satisfaction, self-regulated learning, learning self-efficacy, and IDLE among EFL learners in online learning contexts. This approach imbues the study with cohesion and focus, enabling a more comprehensive understanding of the interconnected constructs.

### 2.2 Online course satisfaction

Satisfaction in education refers to the pleasure derived from achieving desired learning objectives (Elliott and Shin, 2002; Wiers-Jenssen et al., 2002). It represents students’ subjective judgment of the learning process, considering how well the learning environment supports their academic success (Pham et al., 2019; Karbakhsh and Ahmadi Safa, 2020; Ngah et al., 2022). Lim et al. (2020) describe satisfaction as the perceived alignment between students’ expectations and actual outcomes in their educational experience. Studies such as Pangarso and Setyorini (2023) have shown that higher course satisfaction correlates with better mastery of content. Particularly in EFL learning, student satisfaction correlates with achieving language learning goals, thereby enhancing motivation and performance (Rashidi and Moghadam, 2014; Qutob, 2018; Tsai, 2019).

It would also be worthwhile to examine satisfaction in online environments investigation due to its peculiar significance to the motivations and aspirations of learners. It should be bear in mind that student satisfaction with the online course is an integral factor to be delt with for determining quality and effectiveness of an online learning (Elia et al., 2019; Martin and Bolliger, 2022; Torrado and Blanca, 2022). As Moore (2005) ideated, there are five pillars to assess the effectiveness of online learning, namely faculty satisfaction, access, learning effectiveness and cost effectiveness, and more importantly student satisfaction which is conceptualized to be the most important one. Online course satisfaction has a central role in shaping learners’ perceptions regarding instructional quality, as well as their tendencies to participate in future online course learning (Nikou and Maslov, 2023; Paposa and Paposa, 2023). The available evidence suggests that high levels of online satisfaction appear to significantly influence learners’ academic outcomes in the classroom (Abdous, 2019; Daneji et al., 2019; Wu et al., 2022). Unlike students with low online satisfaction, satisfied learners are more likely to be passion and to invest extra effort in online learning. Importantly, high online satisfaction can be significantly conducive to online course completion rates and can encourage learner to be more committed to learning and more motivated to achieve their learning objectives (Kuo et al., 2014; Nashaat et al., 2021; Ranadewa et al., 2021). More particularly, in the context of EFL, Zou et al. (2022) noted that online course satisfaction is positively associated with L2 learners’ adjustment to virtual environment, the utility of the platforms, the experienced enjoyment, the ease of use of the platform, and the effectiveness of the platform.

Previous research across various educational contexts, including EFL learning and teaching, highlights the significance of online course satisfaction. For instance, Jiang et al. (2021) found that perceived usefulness and ease of use significantly influenced satisfaction among 928 Chinese students. Bervell et al. (2020) identified positive correlations between different types of interactions—learner-learner, learner-teacher, and learner-material—and learners’ satisfaction. In the field of SLA, Shih et al. (2013) observed a relationship between online course satisfaction, online learning motivation, and Big Five personality traits in a study involving 53 tertiary-level EFL learners.

Taken together, albeit the bulk of studies that have examined the potential indicators of learners’ online learning satisfaction, relatively fewer studies have touched upon the impact of psychological factors and online learner-related variables on online course satisfaction. Moreover, the exploration of the antecedents of online course satisfaction in the context of EFL is still lagging behind the dynamic sphere and more in-depth qualitative studies are required to increase our understanding of this construct.

### 2.3 Online self-regulated learning

With roots in psychology, self-regulation has been conceptualized by Bandura (1988) as the form of three attributions of cognitive incentives, namely causal ascriptions, outcome expectancies, and cognized objectives. Appropriately, self-regulation has been gradually barrowed from the realm of psychology and has been extended into the educational context, as a result, the concept of self-regulated learning has been introduced for student learning (Jansen et al., 2019; Wolters and Brady, 2020).

In essence, self-regulation entails learners’ kind of process and skills that help them plan and arrange the tasks in the learning context, manage time, ask for support of others (i.e., teachers and peers), and see if the long-term objectives were attained (Posner and Rothbart, 2000; Landrum, 2020). As put forward by Schunk and Zimmerman (2007), self-regulation has to do with learners’ capacity to utilize self-managing behaviors and incorporate learning processes, and to merge these with motivation so that learners could be able to use their self-confidence behaviors in the classroom. According Zimmerman (1989), self-regulated learning pertains to students’ effective engagement within the learning process in four processes of cognitive (i.e., involving the kind of procedure used by learners effectively master the content), metacognitive (i.e., referring to students’ capability to scheme plans, strategies or objectives as an attempt to assess and appraise their own performance and learning), motivational (i.e., focusing on the notion whether students are self-motivated and willing to independently be held as accountable for their own successes or failures), and behavioral (i.e., normally conceived as learners’ seeking help from peers or teachers to facilitate the learning process).

In online environments, students who are equipped with self-regulatory skills have better chances to achieve their goals, as self-regulated learning can significantly improve students’ capacity to exercise self-control, self-monitor and self-evaluate in virtual classrooms (Öztürk and Çakıroğlu, 2021; Lei and Lin, 2022; Saint et al., 2022). To be particular, for EFL learners, self-regulated learning has significant advantages in terms of increasing their self-direction and self-management and encourage them to be independent and autonomous when engaged in online activities (Guo et al., 2021; Teng, 2021; Chen et al., 2023; Shen et al., 2023). As long as L2 learning is considered, given that it can facilitate the dynamic personality of students’ interactions, self-regulated learning is often considered to be one of critical learning strategies for blended learning (Xu et al., 2022).

Previous studies have highlighted the pivotal role of self-regulated learning in enhancing satisfaction and academic outcomes (Kuo et al., 2014; Ejubovic and Puška, 2019; Zalli et al., 2019; Lim et al., 2020; Wu et al., 2023). For instance, Zalli et al. (2019) and Lim et al. (2020) observed a positive impact of self-regulated learning on online course satisfaction. Wu et al. (2023) also identified self-regulated learning as a contributing factor to learners’ satisfaction with online courses. Ejubovic and Puška (2019) discovered a positive association between self-regulated learning, academic performance, and online course satisfaction. However, Landrum (2020) reported conflicting results, as their study did not find self-regulation strategies to significantly predict online learning satisfaction among students.

Despite the crucial role of self-regulated learning in shaping learners’ satisfaction in online environments, there exist scant empirical evidence that delves into association between these two constructs in the realm of SLA. Bearing this in mind, we aimed to investigate the potential role of self-regulated learning in affecting online course satisfaction among EFL learners.

### 2.4 Online learning self-efficacy

Self-efficacy refers to individuals’ beliefs in their ability to perform tasks effectively for desired outcomes (Bandura, 1982). Rooted in social cognitive theory, self-efficacy underscores motivation’s role in learning and performance (Schunk, 1995). It emphasizes how learners acquire beliefs, information, and skills within specific contexts (Al-Abyadh and Abdel Azeem, 2022). People’s self-efficacy significantly influences their actions; those with high self-efficacy are more resilient, less likely to avoid challenges, and perceive difficulties as growth opportunities (Cassidy, 2015; Supervia et al., 2022). In the realm of language learning, EFL learners with higher self-efficacy levels tend to set and persist in achieving demanding goals (Dong et al., 2022; Derakhshan and Fathi, 2023).

#### 2.4.1 Top of form

Like traditional classrooms, self-efficacy has a paramount role in online environments. It is often argued that self-efficacy can play a central role in students’ online learning as it might have great contributions to their success in virtual contexts (Zimmerman and Kulikowich, 2016; Tang et al., 2022). Furthermore, Quang et al. (2022) demonstrated that those EFL learners who are equipped with online self-efficacy tend to be more successful in online environments. More importantly, previous researchers have established that online self-efficacy has been related to students’ wellbeing and success (i.e., online course satisfaction) (e.g., Shen et al., 2013; Suryandani and Santosa, 2021; Aldhahi et al., 2022; Derakhshesh et al., 2022). Aldhahi et al. (2022) aimed to evaluate students’ online learning satisfaction and to examine whether online-learning self-efficacy correlate with satisfaction in remote learning. Collecting data from a total of 1,226 learners, their results revealed that online learning self-efficacy had a positive role in influencing online course satisfaction. In the same vein, Shen et al. (2013) reported that that online learning self-efficacy positively predicted online learning satisfaction among students. The association between online learning self-efficacy and online course satisfaction was the focus of Derakhshesh’s et al. (2022) study. Utilizing structural equation modeling, the authors indicated that online learning self-efficacy could exercise positive effects on online course satisfaction among EFL learners. In a mixed-method research design, Suryandani and Santosa (2021) examined the association between online learning self-efficacy and students’ satisfaction with online courses. The results indicated a positive relationship, suggesting that students’ satisfaction with online courses could be enhanced by their online learning self-efficacy.

What is found from the literature review is that despite a number of research has been done on the interplay between online self-efficacy and online satisfaction in general education context, the study of the potential correlation between these constructs in the EFL classrooms merits further exploration.

### 2.5 IDLE

Informal digital learning of English (IDLE) is a phenomenon which is established in the context of CALL and has recently attracted the attention of the digital-native generation worldwide (Lee and Sylvén, 2021). In fact, IDLE is grounded in incidental language learning (Miller and Godfroid, 2020), and informal language learning (Bahrani and Sim, 2012), and is often recognized as a sub-field of CALL in the context of L2 learning and teaching. Researchers often refer to IDLE as an informal English learning concept that provides a self-directed platform and encompasses different types of computer-based technologies and web-based resources like smart phones, computers, social media, and blogs in an informal context (Lai, 2019; Soyoof et al., 2023). In fact, IDLE is recognized as self-directed and situated in the out-of-class due to the fact that students are the ones that first initiate IDLE activities (Liu et al., 2023). IDLE provides a kind of opportunity by which students can learn a language in an unstructured and naturalistic manner and not in a process in the service of certification (Lee et al., 2023).

It is argued that IDLE can be either form-focused (i.e., merely dealing with linguistic elements and the language accuracy) or meaning-focused (i.e., focusing on the authentic use of language) (Zhang and Liu, 2022). Previous findings from existing literature indicates that IDLE is significantly conducive to contribute L2 learning and teaching. For instance, collecting data from a sample of 1,490 Chinese EFL learners, Liu et al. (2023) revealed that IDLE activities had positive effects on language and culture learning, cultural differences, intercultural communication, and engagement among EFL students. In another study, Lee et al. (2022) found that IDLE could predict students’ willingness to communicate in L2. Notwithstanding these researches, the investigation of the relationship between IDLE and online course satisfaction has remained non-examined in the EFL domain.

Despite the recent attention, the research on IDLE is still very limited and further investigation are required to shed light on the role of this construct in language learning. Furthermore, its correlation with other psychological learner-related variables in online environments has not been examined by the L2 researchers. In fact, albeit the array of research foci, to our knowledge, our research is the first attempt to examine the relations between IDLE and online course satisfaction, online self-regulated learning, and online learning self-efficacy among EFL learners. Consequently, we sought to examine whether IDLE has any significant effects on online course satisfaction via the meditation role of online self-regulated learning in the EFL setting.

### 2.6 The present study

While a significant body of literature has highlighted the positive associations between self-efficacy, informal digital learning, self-regulated learning, and course satisfaction (Lim et al., 2020; Aldhahi et al., 2022; Lee et al., 2023; Wu et al., 2023), the existing research landscape also encompasses contrasting or nuanced findings.

For instance, amidst the consensus on the beneficial impact of self-regulated learning on online course satisfaction, Landrum’s (2020) study revealed contrasting results. Their findings did not identify self-regulation strategies as significant predictors of online learning satisfaction among students. This discrepancy underscores the imperative for deeper explorations into the nuanced aspects of self-regulated learning that might differentially contribute to learners’ course satisfaction.

Moreover, despite considerable evidence supporting the positive impact of IDLE on language proficiency and cultural understanding, certain perspectives highlight potential limitations or contextual dependencies. Zhang and Liu (2022) underscored distinctions between form-focused and meaning-focused IDLE activities, suggesting that their effectiveness might be contingent upon learners’ preferences or specific learning contexts.

Furthermore, within the domain of self-efficacy, while several studies accentuate its positive association with online learning outcomes, a subset of research presents more nuanced perspectives. For instance, Tang et al. (2022) established a significant positive correlation between online self-efficacy and students’ success in online environments, whereas Zimmerman and Kulikowich (2016) observed variations in the impact of self-efficacy based on individual differences and task characteristics.

Acknowledging these divergent or nuanced findings underscores the multifaceted nature of the relationships among self-efficacy, informal digital learning, self-regulated learning, and course satisfaction. The complexity of these relationships seems contingent upon individual differences, contextual factors, and specific learning environments. Thus, comprehensive investigations considering these intricacies are crucial to obtain a deeper understanding of their interplay.

**FIGURE 1:**
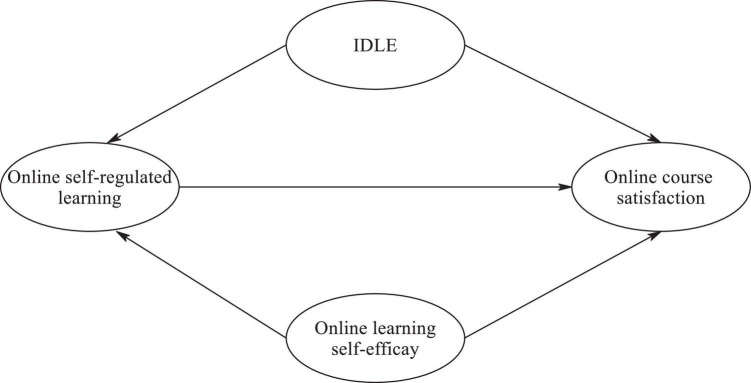
The hypothesized model.

Against this backdrop, the purpose of this study was to empirically test a hypothesized model (see Figure 1) that examines the interrelationships among online learning self-efficacy, informal digital learning of English, online self-regulated learning, and their collective impact on online course satisfaction. The following hypotheses will guide this study:

#### 2.6.1 H1: Online learning self-efficacy directly predicts online course satisfaction

Online learning self-efficacy (OLSE) is concerned with learners’ beliefs in their ability to effectively perform online learning tasks and achieve desired outcomes (Bandura, 1982). OLSE is particularly crucial in shaping learners’ motivation and performance in virtual environments (Zimmerman and Kulikowich, 2016; Tang et al., 2022). Previous research has demonstrated a positive association between OLSE and students’ success and wellbeing in online learning, including online course satisfaction (Shen et al., 2013; Suryandani and Santosa, 2021; Aldhahi et al., 2022; Derakhshesh et al., 2022).

Students with higher OLSE are more likely to set challenging tasks and objectives and persevere in their efforts (Dong et al., 2022). As a result, they are less likely to avoid demanding tasks and view them as opportunities for growth and development (Cassidy, 2015; Supervia et al., 2022). In the context of EFL learners, higher self-efficacy beliefs have been associated with a willingness to engage in challenging language learning activities and a commitment to achieving language proficiency goals (Dong et al., 2022).

#### 2.6.2 H2: Informal digital learning of English directly predicts online course satisfaction

IDLE involves learners’ self-initiated activities using computer-based technologies and web-based resources in an informal context (Lai, 2019; Lee, 2021; Soyoof et al., 2023). Previous research has demonstrated the positive impact of IDLE on language learning outcomes, cultural awareness, and communication skills (Lee and Sylvén, 2021; Lee et al., 2022; Liu et al., 2023). Given the positive associations of IDLE with language learning outcomes and engagement, it is reasonable to hypothesize that learners who engage in IDLE activities may experience higher satisfaction with their online courses.

#### 2.6.3 H3: Online self-regulated learning directly predicts online course satisfaction

Online self-regulated learning (SRL) is rooted in psychology and has been extended into the educational context, particularly in the domain of self-regulated learning (Jansen et al., 2019; Wolters and Brady, 2020; Theobald, 2021). In virtual classrooms, learners need to exercise self-control, self-monitoring, and self-evaluation to effectively engage in the learning process (Öztürk and Çakıroğlu, 2021; Lei and Lin, 2022; Saint et al., 2022). The positive influence of self-regulated learning on online course satisfaction has been observed in various educational contexts (Ejubovic and Puška, 2019; Wu et al., 2023).

#### 2.6.4 H4: Online self-regulated learning acts as a mediating factor in the relationship between OLSE and IDLE, and course satisfaction

Online self-regulation serves as a mediating variable between OLSE, IDLE, and course satisfaction. When learners have higher OLSE and engage in IDLE activities, they are more likely to take a proactive approach to their learning, set challenging goals, and persist in the face of challenges. These behaviors are characteristic of effective self-regulated learners (Zimmerman, 1989). Moreover, previous research has shown that learners with higher OLSE and engagement in IDLE activities are more likely to exhibit stronger self-regulatory skills (Lai, 2019; Liu et al., 2023). Thus, it can be argued that online SRL acts as the mechanism through which OLSE and IDLE influence learners’ satisfaction with online courses.

## 3 Materials and methods

### 3.1 Participants

The study included a diverse sample of 563 intermediate Chinese EFL college students, carefully chosen from multiple national universities across China. Among the participants, there were 247 males (*N* = 247) and 316 females (*N* = 316). The age of the participants ranged from 19 to 26 years, with a mean age of 20.88 years (SD = 2.06). All participants were undergraduate students pursuing various disciplines. Regarding their English proficiency level, the participants were categorized as intermediate. This ensured a homogenous language proficiency level for the study, which allowed for more precise investigations into the relationships between variables. The selection of participants was carried out using a rigorous stratified random sampling technique. Employing this approach, the researchers aimed to achieve comprehensive representation from multiple national universities across diverse geographic regions in China. Stratification based on the geographical distribution of universities was crucial to account for potential regional variations in learning approaches, preferences, and experiences. Consequently, this sampling method minimized biases and enhanced the generalizability of the study’s findings to the broader population of Chinese EFL learners.

The stratified random sampling procedure involved dividing the population of Chinese EFL college students from various national universities into distinct strata based on geographic regions. From each stratum, participants were randomly selected to participate in the research. This approach ensured that every participant had an equal chance of being included in the study, thereby ensuring the validity and representativeness of the findings.

### 3.2 Instruments

Four assessment instruments were employed in this study to measure various aspects of participants’ experiences with online learning (see Supplementary Appendix). The first instrument, the Online Course Satisfaction Scale (OCSS), developed by Wei and Chou (2020), consists of 7 items assessing learners’ satisfaction with an online course. These items gauge perceptions regarding course design and teachers’ performance. Participants indicated their responses on a 5-point Likert scale, ranging from strongly disagree (1) to strongly agree (5).

For assessing informal digital English learning, the IDLE Scale was adapted from Lee and Drajati’s (2019) original version. The scale includes four subscales: form-focused activities (FF), game-based activities (GB), receptive IDLE activities (RE), and productive IDLE activities (PR). Participants reported their engagement frequency in these activities on a 5-point Likert-type scale, varying from never (1) to very often—many times per day (5). A sample item included, “I use Google to check grammar and vocabulary.”

To measure online learner self-regulation, the Online Self-Regulated Learning Questionnaire (OSLQ) was employed, based on Barnard et al.’s (2010) work. The OSLQ consists of 24 items and assesses constructs such as goal setting, time management, help seeking, task strategies, and self-evaluation. Participants responded to statements using a five-point Likert-type scale, ranging from strongly disagree to strongly agree. The OSLQ comprises six subscales, each reflecting different aspects of self-regulation. Reliability coefficients for the subscales were reported to be 0.80 and above (Barnard et al., 2009).

Lastly, the online learning self-efficacy scale (OLSS) by Tsai et al. (2020) was utilized, featuring 25 items assessing five distinct aspects of self-efficacy related to online learning. These aspects include participants’ confidence in successfully completing an online course, engaging socially with peers, navigating Course Management System (CMS) tools, interacting with online instructors, and collaborating with classmates for academic purposes. Participants rated their agreement with each statement using a 5-point Likert scale, ranging from strongly disagree (1) to strongly agree (5).

### 3.3 Procedure

Participants were recruited through official communication channels of their respective national universities. Before data collection, the researchers sought ethical approval from the institutional review board at their affiliated university. Eligible participants received a formal invitation, which detailed the purpose, significance, and confidentiality of the study. Emphasis was placed on voluntary participation and informed consent, with the assurance that their involvement would not impact their academic standing or any other aspect of their university life. Data collection occurred through an online survey questionnaire hosted on a secure and confidential platform. The survey period extended over 5 weeks, allowing participants sufficient time to complete the questionnaire at their convenience. Regular reminders were sent to encourage participation and maximize response rates. Informed consent was obtained from all participants, ensuring they were fully aware of the confidentiality and anonymity of their responses. To protect participants’ privacy, personal identifiers were removed from the dataset. The research strictly adhered to the guidelines and principles outlined in the Declaration of Helsinki, ensuring the rights and welfare of the participants were safeguarded throughout the study. For data collection, a secure and confidential online survey platform was selected. This platform was chosen for its user-friendly interface, accessibility, and robust data management capabilities. Adhering to industry standards, the survey platform implemented data encryption and protection measures to ensure the confidentiality and integrity of participants’ responses.

### 3.4 Data analysis

To analyze the data, descriptive statistics and correlations were computed using SPSS 28.0. Subsequently, confirmatory factor analysis (CFA) was performed through AMOS 26.0, a software application commonly utilized for Covariance-Based Structural Equation Modeling (CB-SEM) (Sarstedt et al., 2022).

We employed AMOS for effective assessment of construct validity and model fit, followed by SEM to explore hypothesized relationships between latent constructs. The choice of AMOS in our analysis stems from its advantages in the context of CB-SEM, providing a robust platform for evaluating complex relationships in our research framework. Notably, CB-SEM, distinct from partial least squares (PLS)-SEM, focuses on explaining covariances between observed variables and latent constructs, making it suitable for theory-driven models like ours (Hair et al., 2006; Dash and Paul, 2021). This aligns with our objective of elucidating interrelationships between self-efficacy, informal digital learning, self-regulated learning, and course satisfaction constructs.

To evaluate the adequacy of the model, widely recognized fit measures were employed, in accordance with the guidelines suggested by Hu and Bentler (1999). These fit measures encompassed the ratio of χ2 goodness-of-fit to degrees of freedom (df), the goodness of fit index (GFI), the comparative fit index (CFI), the Root-Mean-Square Error of Approximation (RMSEA), and the Standardized Root-Mean-Square Residual (SRMR). A χ2/df ratio below 3, accompanied by a *p*-value exceeding 0.05, indicated a good fit. Additionally, GFI and CFI values equal to or greater than 0.90 were considered indicative of a good fit, while RMSEA values below 0.08 and SRMR values below 0.10 were considered acceptable fit. To assess the significance of the indirect effects, bootstrapping analyses with 5,000 resamples were conducted, following Hayes’ (2013) approach.

## 4 Results

Prior to conducting substantive analyses, we conducted thorough data quality checks to ensure the integrity and reliability of our dataset. These checks encompassed a series of procedures aimed at identifying and addressing potential issues related to data completeness, accuracy, and consistency.

First, we examined missing data patterns across all variables included in the study. The analysis revealed that less than 5% of the data were missing, which were predominantly due to occasional non-responses by participants. We employed a principled approach to handle missing data, utilizing listwise deletion for cases with missing values, given the relatively low rate of non-response (Kline, 2023). Sensitivity analyses were conducted to evaluate the impact of missing data on our findings, confirming that the final sample remained representative of the original cohort.

Next, we assessed the distributional properties of the variables, examining for outliers, skewness, and kurtosis. Outliers were defined as values falling more than 1.5 times the interquartile range above the third quartile or below the first quartile (Kline, 2023). In our dataset, we observed a minimal number of outliers (less than 2% of cases), which were retained in the analysis as they did not exert undue influence on the results.

Additionally, we conducted a normality test on each variable item, following the approach outlined (Hair et al., 2006). The results, displayed in Table 4, reveal that the skewness values fall within the range of −0.19 to −0.08, indicating a relative proximity to 0 and suggesting a symmetric distribution of the data. Moreover, the kurtosis values range from 0.14 to 0.21, which are in close approximation to the expected value of 3, signifying that the data exhibits relatively normal tail behavior. Based on these observations, it is reasonable to conclude that our dataset demonstrates an approximate normal distribution, given the proximity of skewness and kurtosis values to 0 and 3, respectively.

Furthermore, we examined the consistency and reliability of the measures through Cronbach’s alpha coefficients. All constructs demonstrated high internal consistency, with values exceeding the recommended threshold of 0.7 (Hair et al., 2006), affirming the reliability of our measurement model. Lastly, we conducted preliminary assessments to identify potential sources of non-response bias. We compared demographic characteristics, such as age, gender, educational background, and prior online learning experience, and key study variables between respondents and non-respondents, finding no substantial differences that would impede the generalizability of our findings. Additionally, we examined the response rates and patterns across different survey items and found no substantial variation that could indicate significant non-response bias.

Then, CFA was conducted using AMOS 26.0 to establish the validity and robustness of the measurement model. The analysis aimed to evaluate the proposed relationships between the latent constructs and their observed indicators and to assess the overall fit of the model to the data. The initial measurement model included indicators for each of the four latent constructs, with multiple observed indicators derived from their respective scales. However, the initial model did not meet the criteria for a satisfactory fit, as indicated by the following fit indices: χ^2^(259) = 573.621, CFI = 0.901, Tucker-Lewis Index (TLI) = 0.898, RMSEA = 0.086, SRMR = 0.074.

To enhance the construct validity and improve model fit, an iterative refinement process was undertaken. This process involved meticulous examination of modification indices and adherence to established guidelines to identify potential sources of model misfit. Consequently, two items from the online learning self-efficacy scale (OLSS7 and OLSS19) were identified as having low factor loadings, indicating inadequate representation of the underlying construct. As a result, these items were removed from the model to strengthen the construct’s validity and conceptual coherence.

Similarly, during the refinement process, two items from the online self-regulated learning scale (OSLQ3 and OSLQ16) and one item each from the IDLE (IDLE10) and online course satisfaction (OCSS3) scales exhibited weak factor loadings. In response, these items were excluded from the model to enhance the clarity and unidimensionality of their respective constructs. Following these modifications, the revised measurement model underwent another round of CFA analysis, demonstrating a substantial improvement in fit indices. The fit indices for the revised model were as follows: χ^2^(281) = 524.389, CFI = 0.953, TLI = 0.958, RMSEA = 0.039, and SRMR = 0.046.

Comparing the fit indices between the initial and revised models unequivocally reveals that the revised model exhibited significantly better goodness-of-fit statistics. Based on the considerable improvement in fit indices and the enhanced construct validity achieved through the iterative refinement process, the revised model was deemed suitable for subsequent data analyses. Table 1 indicates the standardized loadings and *t*-values of the observed indicators on their respective latent constructs. The factor loadings of all indicators were significant (*p* < 0.001).

**TABLE 1:** The results of measurement model.

Constructs	Indicators	Standardized loading	*t*-value
OCSS	OCSS1	0.75	6.43
	OCSS2	0.81	7.29
	OCSS4	0.72	6.12
	OCSS5	0.76	6.57
	OCSS6	0.70	5.95
	OCSS7	0.79	7.03
IDLE	IDLE1	0.64	4.89
	IDLE2	0.72	5.95
	IDLE3	0.68	5.22
	IDLE4	0.71	5.75
	IDLE5	0.69	5.45
	IDLE6	0.75	6.32
	IDLE7	0.70	5.62
	IDLE8	0.67	5.13
	IDLE9	0.71	5.79
	IDLE11	0.66	4.98
	IDLE12	0.70	5.60
	IDLE13	0.73	6.04
OSLQ	OSLQ1	0.80	8.12
	OSLQ2	0.79	7.98
	OSLQ4	0.76	7.32
	OSLQ5	0.81	8.03
	OSLQ6	0.78	7.69
	OSLQ7	0.84	8.95
	OSLQ8	0.79	7.92
	OSLQ9	0.82	8.54
	OSLQ10	0.77	7.41
	OSLQ11	0.80	8.21
	OSLQ12	0.75	7.18
	OSLQ13	0.83	8.71
	OSLQ14	0.78	7.63
	OSLQ15	0.81	8.07
	OSLQ17	0.80	8.28
	OSLQ18	0.82	8.63
	OSLQ19	0.75	7.15
	OSLQ20	0.81	8.01
	OSLQ21	0.76	7.28
	OSLQ22	0.83	8.69
	OSLQ23	0.79	7.85
	OSLQ24	0.82	8.58
OLSS	OLSS1	0.70	6.02
	OLSS2	0.68	5.48
	OLSS3	0.71	6.16
	OLSS4	0.75	6.71
	OLSS5	0.73	6.32
	OLSS6	0.69	5.76
	OLSS8	0.72	6.19
	OLSS9	0.70	5.93
	OLSS10	0.68	5.43
	OLSS11	0.75	6.63
	OLSS12	0.73	6.28
	OLSS13	0.71	6.07
	OLSS14	0.74	6.38
	OLSS15	0.70	5.85
	OLSS16	0.68	5.39
	OLSS17	0.75	6.58
	OLSS18	0.73	6.25
	OLSS20	0.74	6.40
	OLSS21	0.70	5.90
	OLSS22	0.68	5.54
	OLSS23	0.75	6.65
	OLSS24	0.73	6.27
	OLSS25	0.71	6.04

OCSS, online course satisfaction; OSLQ, online self-regulated learning; OLSS, online learning self-efficacy.

Then we examined convergent and divergent validity of the latent variables in the measurement model (see Table 2). Convergent validity indicates the extent to which indicators of a latent construct are related, while divergent validity assesses the distinctiveness of different constructs (Fornell and Larcker, 1981).

**TABLE 2:** Convergent and divergent validity.

Variables	AVE	CR	1	2	3	4
1. Online self-efficacy	0.56	0.91	*0.74*			
2. IDLE	0.62	0.85	0.33**	*0.78*		
3. Online self-regulation	0.73	0.87	0.40***	0.41***	*0.85*	
4. Online satisfaction	0.67	0.81	0.44***	0.27*	0.51***	*0.81*

Online satisfaction, online course satisfaction; AVE, average variance extracted; CR, composite reliability. Italic font numbers are square roots of the AVE; off diagonals are correlation confidents;

*p < 0.05;

**p < 0.01;

***p < 0.001.

The average variance extracted (AVE) and composite reliability (CR) were calculated to assess convergent validity. The square roots of the AVE are presented in italic font in Table 2, and the correlation coefficients are displayed off the diagonals. The correlations between different constructs provide evidence of divergent validity. The correlation coefficients are within an acceptable range, suggesting that the latent constructs are distinct from each other.

In addition to the Fornell–Larcker test, it is advisable to incorporate the evaluation of HTMT (heterotrait–monotrait ratio of correlations) values to conduct a thorough assessment of discriminant validity (Hair et al., 2006; Wijaya et al., 2022). When employing the HTMT criterion, a construct is considered to have strong discriminant validity if the HTMT value remains below the 0.9 threshold. This approach serves to confirm the discriminant validity in our study, as evident from the results presented in Table 3.

**TABLE 3:** HTMT values.

	1	2	3	4
1. Online self-efficacy	–			
2. IDLE	0.33	–		
3. Online self-regulation	0.40	0.41	–	
4. Online satisfaction	0.44	0.27	0.51	–

Overall, these results affirm the measurement model’s convergent and divergent validity, indicating that the indicators of each latent construct are related, while the constructs themselves are distinguishable. The analysis supports the suitability of the measurement model for subsequent analyses and confirms the validity of the conceptual framework used to explore the relationships between the constructs in EFL learners.

Table 4 presents the descriptive statistics and reliability indices for the measured variables in the study. Participants reported a mean score of 3.81 (SD = 0.73) on the online learning self-efficacy scale, indicating a moderate level of self-efficacy in online learning. The internal consistency of the scale was found to be satisfactory (Cronbach’s α = 0.81), indicating good reliability. For IDLE, participants had a mean score of 3.09 (SD = 0.95), suggesting a moderate level of engagement in informal digital learning activities for English. The internal consistency of the IDLE scale was high (Cronbach’s α = 0.87), indicating strong reliability.

**TABLE 4:** Descriptive statistics and reliability.

Variables	Mean	SD	Skewness	Kurtosis	Cronbach’s alpha
1. Online self-efficacy	3.81	0.73	−0.19	0.21	0.81
2. IDLE	3.09	0.95	−0.11	0.20	0.87
3. Online self-regulation	3.66	0.77	−0.08	0.19	0.92
4. Online satisfaction	3.88	0.69	−0.06	0.14	0.84

Regarding online self-regulation, the students reported a mean score of 3.66 (SD = 0.77), indicating a moderate level of self-regulated learning in the online context. The internal consistency of the scale was excellent (Cronbach’s α = 0.92), indicating high reliability. In terms of online course satisfaction, participants had a mean score of 3.88 (SD = 0.69), suggesting a relatively high level of satisfaction with online learning activities. The internal consistency of the scale was found to be satisfactory (Cronbach’s α = 0.84), indicating good reliability.

Then, the correlations among the constructs were calculated (see Table 5). As seen in Table 5, online self-efficacy is positively and significantly correlated with online self-regulation (*r* = 0.40, *p* < 0.001) and online course satisfaction (*r* = 0.44, *p* < 0.001). This indicates that higher levels of self-efficacy in online learning are associated with higher levels of online self-regulated learning and greater satisfaction with the online course. IDLE exhibits a positive and significant correlation with online self-regulation (*r* = 0.41, *p* < 0.001) and online course satisfaction (*r* = 0.27, *p* < 0.05). This suggests that a higher level of informal digital learning engagement in English is related to increased levels of online self-regulated learning and a higher degree of satisfaction with the online course. Online self-regulation is significantly and positively correlated with online course satisfaction (*r* = 0.51, *p* < 0.001). This indicates that a greater degree of self-regulated learning in the online context is associated with increased satisfaction with the online course. These findings highlight the interrelated nature of the constructs under investigation. Specifically, the positive correlations between online self-efficacy, IDLE, online self-regulation, and online course satisfaction support the hypothesized relationships posited in the conceptual framework. This suggests that learners who perceive themselves as efficacious in online learning and engage in informal digital learning activities in English are more likely to demonstrate self-regulated learning behaviors in the online course and experience higher levels of satisfaction with the learning experience.

**TABLE 5:** Correlations among the constructs.

Variables	1	2	3	4
1. Online self-efficacy	1.00			
2. IDLE	0.33**	1.00		
3. Online self-regulation	0.40***	0.41***	1.00	
4. Online satisfaction	0.44***	0.27*	0.51***	1.00

*p < 0.05,

**p < 0.01,

***p < 0.001.

The significant correlations observed between the latent variables lend support to the validity of the measurement model and provide evidence for the relationships postulated in the research hypotheses. The findings contribute to a better understanding of the complex interplay between online learning self-efficacy, IDLE, online self-regulated learning, and online course satisfaction in the context of EFL learners’ online courses.

**FIGURE 2:**
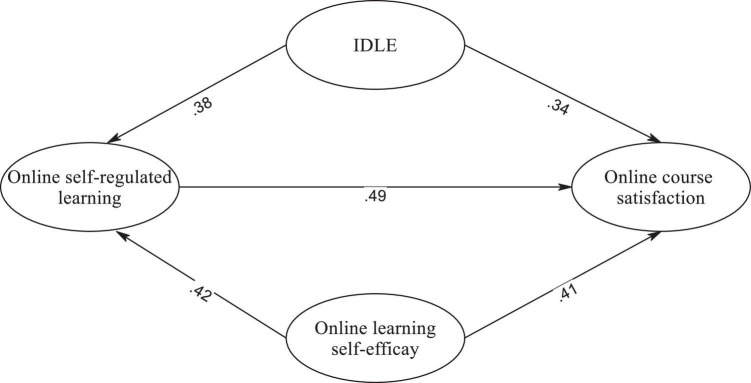
The structural equation modeling (SEM) model. The relationships between variables depicted in the model are significant at the *p* < 0.05 level.

Structural equation modeling (SEM) was utilized to investigate the hypothesized relationships between the latent constructs. The model fit indices indicated a satisfactory fit to the data: χ^2^(362) = 672.452, *p* = 0.000, CFI = 0.952, TLI = 0.948, and RMSEA = 0.041, 95% CI = 0.036–0.047. These indices collectively suggest that the proposed structural model adequately represents the relationships between the latent constructs. Figure 2 depicts the path diagram illustrating the hypothesized relationships between the constructs. All path coefficients were found to be statistically significant. The specific path coefficients are as follows: IDLE positively and significantly predicted online course satisfaction (β = 0.34). Learners who engage more in informal digital learning activities for English tend to exhibit higher levels of satisfaction in online learning.

Online self-efficacy positively and significantly influenced online course satisfaction (β = 0.41). Learners’ beliefs in their ability to perform well in online learning are associated with higher levels of satisfaction with the online course. Online self-regulated learning had a positive and significant effect on online course satisfaction (β = 0.49). Learners who demonstrate better self-regulated learning behaviors in the online context are more satisfied with online learning activities in the course. IDLE positively and significantly predicted online self-regulated learning (β = 0.38). Learners who engage more in informal digital learning activities for English tend to demonstrate higher levels of self-regulated learning in the online environment. Online learning self-efficacy had a positive and significant influence on online self-regulated learning (β = 0.42). Learners with higher levels of self-efficacy in online learning are more likely to exhibit better self-regulated learning behaviors in the online context.

These results provide empirical support for the hypothesized relationships between the constructs and highlight the significant role of IDLE, online self-efficacy, and online self-regulated learning in predicting learners’ satisfaction with the online course. The findings contribute to a deeper understanding of the factors influencing learners’ satisfaction in online learning environments and hold practical implications for EFL educators and instructional designers seeking to enhance the quality of online courses.

To evaluate the significance of the indirect effects, bootstrapping analyses were conducted using 5,000 resamples (Hayes, 2013). Table 6 summarizes the results of the bootstrapping analyses, presenting the direct, indirect, and total effects in the mediation analysis. The direct effects between the predictor variables and the outcome variable were statistically significant. Specifically, online self-efficacy (β = 0.41, *p* < 0.001), IDLE (β = 0.34, *p* < 0.001), and online self-regulation (β = 0.49, *p* < 0.001) had positive and significant relationships with online course satisfaction. The mediation analysis further revealed significant indirect effects. Online self-efficacy showed a significant indirect effect on online course satisfaction through online self-regulated learning (β = 0.20, 95% CI [0.14, 0.26], *p* < 0.001). Similarly, IDLE had a significant indirect effect on online course satisfaction through online self-regulated learning (β = 0.18, 95% CI [0.12, 0.24], *p* < 0.001).

**TABLE 6:** The results of SEM.

Path	Coefficient (β)	Bootstrapped 95% CI	*p*-value	Effect
Online self-efficacy → online satisfaction	0.41	[0.35, 0.48]	< 0.001	Direct
IDLE → online satisfaction	0.34	[0.27, 0.41]	< 0.001	Direct
Online self-regulation → online satisfaction	0.49	[0.42, 0.56]	< 0.001	Direct
Online self-efficacy → OSRL → online satisfaction	0.20	[0.14, 0.26]	< 0.001	Indirect
IDLE → OSRL → online satisfaction	0.18	[0.12, 0.24]	< 0.001	Indirect
Online self-efficacy → online satisfaction	0.61	[0.55, 0.67]	< 0.001	Total
IDLE → online satisfaction	0.52	[0.46, 0.58]	< 0.001	Total

OSRL, online self-regulated learning; online satisfaction, online course satisfaction.

Furthermore, the total effects, which combine the direct and indirect effects, were also statistically significant. Online self-efficacy had a total effect of 0.61 (95% CI [0.55, 0.67], *p* < 0.001) on online course satisfaction, while IDLE had a total effect of 0.52 (95% CI [0.46, 0.58], *p* < 0.001).

Furthermore, we employed Harman’s single-factor test, a well-recognized technique for assessing common method bias (Podsakoff et al., 2003). In this test, we conducted an exploratory factor analysis (EFA) on all items included in the study, with all items loaded onto a single factor. If common method bias was a significant concern, a single factor would account for a substantial proportion of the variance. However, the results of the EFA showed that the single factor accounted for 27.36% of the variance, suggesting that common method bias is unlikely to be a major issue in our dataset.

In addition, we performed a common latent factor analysis, following the procedure suggested by Kock and Lynn (2012). This analysis examines whether a common latent factor accounts for the covariance among the variables. The results indicated that a common latent factor did not significantly account for the variance in the observed variables, further supporting the absence of substantial common method bias.

Overall, the mediation analysis provided valuable insights into the underlying mechanisms through which online learning self-efficacy and IDLE influence online course satisfaction. The results indicate that both online self-efficacy and IDLE have direct effects on online course satisfaction, and these effects are partially mediated by online self-regulated learning. These findings contribute to a better understanding of the factors influencing online learning satisfaction in EFL learners and highlight the significance of self-efficacy and informal digital learning experiences in fostering online course satisfaction and self-regulated learning behaviors in the online learning context. The findings have practical implications for educators and course designers seeking to optimize the online learning experience and enhance learner engagement and satisfaction in EFL contexts.

## 5 Discussion

In this research we sought to investigate a predictive role of online learning self-efficacy, self-regulated learning, and IDLE on online course satisfaction among EFL learner in the context of China. By doing this, the current study can offer a diverse range of both theoretical and pedagogical implications for EFL learning and teaching.

First, the findings indicated that online learning self-efficacy had a positive and direct impact on online course satisfaction. Our findings align with prior research that has indicated a positive relationship between online learning self-efficacy and learners’ satisfaction with online courses (e.g., Shen et al., 2013; Suryandani and Santosa, 2021; Aldhahi et al., 2022; Derakhshesh et al., 2022). This finding supports Bandura’s social cognitive theory, which posits that individuals’ beliefs in their own ability to perform tasks and achieve desired outcomes play a crucial role in shaping their motivation and performance (Bandura, 1982, 1997). The positive influence of online learning self-efficacy on online course satisfaction can be explained by the role of self-efficacy in reducing learners’ anxiety and stress in virtual learning environments (Passiatore et al., 2019; Ferreira et al., 2020). In online settings, students may face unique challenges and uncertainties, and higher levels of self-efficacy can act as a buffer against the negative effects of anxiety and stress. As a result, learners with higher self-efficacy beliefs are more likely to feel confident and comfortable engaging in online activities, which in turn enhances their satisfaction and enjoyment in the learning process (Bolliger and Halupa, 2012; Kuo et al., 2014).

Furthermore, students with higher online learning self-efficacy are more inclined to set challenging tasks and goals, persevere in their efforts, and view difficulties as opportunities for growth (Dong et al., 2022). These self-directed and proactive behaviors are characteristic of effective self-regulated learners, which, in turn, can contribute to higher satisfaction with online courses (Zimmerman, 1989). When learners believe in their ability to succeed, they are more likely to take charge of their learning process, seek resources, and actively engage with the course materials, leading to a more fulfilling learning experience (Ji et al., 2022). As Landrum (2020) added, students’ self-efficacy in virtual classes can be greatly conducive to students’ course satisfaction of online learning.

Second, the findings of this study indicate that online self-regulated learning plays a significant and positive role in predicting online course satisfaction among EFL students. This finding is in line with previous research in the field of general education, which has also demonstrated the positive impact of self-regulated learning on learners’ course satisfaction (Kuo et al., 2014; Ejubovic and Puška, 2019; Zalli et al., 2019; Lim et al., 2020; Wu et al., 2023). The ability of learners to regulate their learning process, plan their approach, and seek support from teachers and peers contributes to their resilience in the face of setbacks and challenges commonly encountered in online learning environments, leading to a higher level of satisfaction with the online course. Self-regulated learners demonstrate certain key characteristics that enhance their learning experience (Theobald, 2021) and, consequently, their satisfaction with online courses. Firstly, they are skilled in setting clear and achievable goals and objectives (Teng, 2021), which fosters their focus and motivation throughout the duration of the online course (Zimmerman, 1989, 1990). Having a sense of purpose and direction in their learning journey enables EFL students to approach online learning with determination and purpose, which in turn enhances their satisfaction with the progress they make and the achievements they attain (Kuo et al., 2014; Lim et al., 2020; Saint et al., 2022).

Moreover, self-regulated learners proactively employ various learning strategies to enhance their understanding and mastery of the course materials (Zimmerman, 2002). Engaging in activities such as seeking additional resources, organizing study materials, and actively participating in discussions enables them to take a more active role in their learning process. These proactive behaviors contribute to a more fulfilling and rewarding learning experience, resulting in higher levels of satisfaction with the online course. Additionally, online self-regulated learners take responsibility for managing their time and learning pace (Zimmerman and Kulikowich, 2016; Zalli et al., 2019; Xu et al., 2022). Their ability to function effectively with less dependence on external structures and guidance allows them to exercise autonomy in their learning journey. This autonomy and flexibility in online learning empower learners as they feel more in control of their learning process, leading to a heightened sense of satisfaction.

Third, it was revealed that IDLE directly predicted online course satisfaction. This result underscores the critical role that self-initiated digital language learning activities play in shaping learners’ satisfaction within online course environments. Numerous studies have recognized the efficacy of IDLE in fostering language learning outcomes, cultural understanding, and communication skills (Lee and Sylvén, 2021; Lee et al., 2022; Liu et al., 2023). These findings have consistently highlighted the positive impact of IDLE on learners’ engagement and language acquisition processes.

The positive association between IDLE and online course satisfaction can be comprehensively understood through several interrelated factors supported by previous literature. Firstly, IDLE often allows learners to take charge of their language learning process in an informal context (Lai, 2019; Lee, 2021). This autonomous engagement empowers learners, providing them with a sense of control over their learning process, which positively impacts their satisfaction within online courses (Lee, 2019, 2021; Lee and Drajati, 2019). Secondly, the diverse range of resources available in IDLE, such as digital platforms, social media, blogs, and various computer-based technologies, offers learners opportunities to engage with language learning in a more naturalistic and unstructured manner (Lee et al., 2023; Soyoof et al., 2023). Such unstructured, self-directed learning experiences have been associated with increased motivation and interest among learners, which can contribute significantly to their satisfaction with the overall learning experience (Liu et al., 2023).

Furthermore, IDLE often bridges the gap between formal learning contexts and real-world applications, allowing learners to practice language skills in authentic situations (Lee and Sylvén, 2021). This practical, real-life application can enhance learners’ confidence in using the language, thereby positively impacting their satisfaction with online language courses. The direct prediction of online course satisfaction by IDLE also underscores the evolving nature of language learning in digital environments (Lee and Drajati, 2019; Lee, 2021). In the contemporary educational landscape, where technology is ubiquitous, IDLE represents a flexible and accessible avenue for learners to supplement their formal learning experiences (Lee et al., 2022). This flexibility and accessibility contribute to learners’ overall satisfaction, as they are able to tailor their language learning experiences to suit their preferences and needs.

Finally, the present research has shed light on the mediating role of online self-regulated learning in the relationship between IDLE, online learning self-efficacy, and online course satisfaction among EFL learners. The findings suggest that higher levels of online learning self-efficacy are associated with a greater propensity for engaging in self-regulated learning behaviors. Learners who possess strong self-efficacy beliefs in their ability to manage their learning effectively are empowered to set challenging goals, apply effort, and persist in their learning activities (Zimmerman, 2000; Jansen et al., 2019; Dinh and Nguyen, 2022). As a result, these self-efficacious learners are more likely to adopt effective learning strategies and utilize available resources optimally, leading to enhanced engagement and meaningful interactions with course materials, instructors, and peers. This heightened engagement, in turn, contributes to their overall satisfaction with the online learning experience (Kuo et al., 2014).

Similarly, IDLE activities, being learner-centered, provide EFL learners with a sense of autonomy and responsibility over their own learning process (Lee, 2021). Consequently, it is reasonable to assume that engagement in IDLE may lead to greater online course satisfaction among EFL students. Indeed, studies have shown that autonomy can positively impact learners’ sense of satisfaction in online environments (Mohammadi Zenouzagh et al., 2023). Furthermore, IDLE activities can foster the development of learners’ social skills through increased interaction opportunities with others on interactive platforms, potentially boosting online course satisfaction. Moreover, learners who actively engage in IDLE experiences are likely to apply self-regulated learning strategies to effectively manage their informal learning activities. Such engagement in IDLE encourages learners to take responsibility for their learning, set goals, and monitor their progress, all of which align with the principles of self-regulated learning (Zhang and Liu, 2022; Soyoof et al., 2023). As learners apply self-regulated learning strategies in their IDLE experiences, they develop a strong foundation of self-regulatory skills that can extend to their formal online courses (Lee, 2021).

Furthermore, the role of online self-regulated learning in shaping learners’ responses to challenges and setbacks during their online language learning journey cannot be overlooked. Self-regulated learners are more likely to perceive obstacles as surmountable and persist in the face of difficulties, resulting in a more positive emotional experience during online learning (Bandura, 1988). This positive emotional experience, in turn, contributes to their overall satisfaction with the course.

Additionally, the findings of this study align closely with the principles of SDT introduced by Deci and Ryan (1985). SDT posits that individuals harbor inherent psychological needs for autonomy, competence, and relatedness. When these fundamental needs find fulfillment, individuals are more inclined to willingly engage in activities and experience heightened satisfaction (Ryan and Deci, 2000). The SEM analysis conducted in this study unveiled direct and positive effects of both online learning self-efficacy and IDLE on online course satisfaction. This discovery implies that when learners possess belief in their capacity to excel in online learning and actively partake in informal digital language learning experiences that extend beyond the formal curriculum, they are more likely to find contentment with their online courses (Hsu et al., 2019).

From the vantage point of SDT, this heightened confidence among learners amplifies their sense of autonomy and competence, which, as per self-determination theory, stand as pivotal psychological needs (Deci and Ryan, 2015). This cultivated sense of autonomy and competence acts as a catalyst for elevated satisfaction with online courses. Furthermore, the involvement in informal digital language learning experiences beyond the prescribed curriculum serves as a means to gratify learners’ need for autonomy and competence (Chen and Jang, 2010). Actively seeking supplementary opportunities for language learning beyond the obligatory coursework demonstrates autonomy, as learners take the reins of their own educational journey. This active engagement not only bestows them with a profound sense of autonomy but also propels the development of their language skills, ultimately heightening their perceived competence (Huang et al., 2019).

Moreover, the study divulged that online self-regulated learning operates as a partial mediator between online learning self-efficacy/IDLE and online course satisfaction. This observation mirrors the tenets of self-determination theory. Self-regulated learning showcases learners’ ability to autonomously oversee and direct their learning process. Through the establishment of objectives and continuous monitoring of progress, learners wield control over their education, thereby satiating their need for autonomy (Deci and Ryan, 2000). Additionally, the deployment of effective learning strategies not only underscores learners’ competence in navigating online course materials effectively (Roca and Gagné, 2008; Chiu, 2022), but also fortifies their sense of autonomy and competence, thus further heightening their satisfaction levels with online courses.

Although our study highlights the positive relationships between online learning self-efficacy, self-regulated learning, IDLE, and course satisfaction, it is crucial to consider alternative factors that might influence these results. One such factor could be the diverse nature of learners’ prior experiences and skills in online learning environments. Individual differences, such as technological proficiency, prior exposure to online courses, or varying degrees of digital literacy, could significantly impact how learners engage with self-regulated strategies, IDLE activities, and ultimately perceive their efficacy in online learning contexts (Zimmerman and Kulikowich, 2016; Tang et al., 2022).

Furthermore, contextual aspects, including the design and structure of online courses, teaching methodologies, and the availability of resources and support, may also contribute to the observed relationships. For instance, certain course designs might favor or hinder the application of self-regulated learning strategies, impacting learners’ satisfaction with the learning process (Landrum, 2020). Additionally, variations in the quality and type of IDLE activities or the accessibility of digital resources might influence learners’ experiences and subsequently, their satisfaction levels with the online course (Lee et al., 2022; Liu et al., 2023).

Another plausible explanation could involve the dynamic nature of motivation and engagement in online learning. While our study emphasizes the positive associations between self-regulation, IDLE, and course satisfaction, other motivational factors, such as intrinsic motivation, interest in the subject matter, or social interaction within the virtual classroom, might interact with or moderate these relationships (Deci and Ryan, 1985; Lee and Sylvén, 2021; Liu et al., 2023). By acknowledging these potential alternative explanations, we recognize the complexity of the relationships investigated in our study. Future research could delve deeper into these factors to unravel their specific contributions and interactions, providing a more comprehensive understanding of the dynamics influencing online course satisfaction among EFL learners.

## 6 Conclusion

The present research investigated the intricate interrelationships between online learning self-efficacy, IDLE, online self-regulated learning, and online course satisfaction among EFL learners in the milieu of online language learning. The outcomes of this study reveal that online learning self-efficacy and IDLE exert direct and positive effects on online course satisfaction among EFL learners. Learners’ confidence in their online learning competencies and their engagement in informal digital language learning experiences play pivotal roles in shaping their satisfaction with online courses. Additionally, online self-regulation has been identified as a significant mediator, highlighting the importance of learners’ self-regulatory behaviors in influencing the impact of self-efficacy and IDLE on course satisfaction.

Overall, these findings support the principles of self-determination theory (Deci and Ryan, 1985) by highlighting the importance of fulfilling psychological needs for autonomy and competence in promoting satisfaction with online courses. When learners believe in their abilities (online learning self-efficacy), engage in IDLE, and actively regulate their own learning process (self-regulated learning), they are more likely to experience higher levels of satisfaction with online courses.

## 7 Implications

The findings of this study make valuable contributions to our theoretical understanding of online learning, shedding light on the pivotal roles played by online learning self-efficacy, IDLE, and online self-regulation in shaping learners’ satisfaction with online courses. This study underscores the need to consider not just the content and design of courses, but also the psychological and self-regulatory factors of learners, all of which are critical in promoting a successful online learning experience. This deeper insight enriches the evolving field of online education research and strengthens the theoretical foundations for comprehending learner satisfaction in virtual learning environments.

On a practical note, the implications of this research carry significant weight for educators, institutions, EFL stakeholders, and policymakers. The findings underscore the pivotal role of online learning self-efficacy in influencing course satisfaction. Educators and institutions should prioritize efforts to nurture learners’ online learning self-efficacy. This can be achieved by providing learners with opportunities for success and by offering constructive feedback. By instilling a sense of efficacy in learners, educators can motivate them to tackle challenges, persist in their learning activities, and ultimately elevate their satisfaction with online courses.

It is important to note that increased satisfaction has been associated with more time spent online, improved grades, and overall academic success in L2 learning and teaching (Amoush and Mizher, 2023). Therefore, EFL stakeholders and policymakers should pay greater attention to this construct, striving to encourage friendly interactions among students that can enhance online learning self-efficacy and satisfaction within virtual environments. This study also underscores the significance of creating user-friendly online learning platforms that provide students with comfort, enjoyment, and reduced anxiety and stress. Such platforms are more likely to result in student satisfaction and, consequently, better L2 acquisition (Fathi and Mohammaddokht, 2021). Institutions should consider these implications when designing online language courses, as interactive and engaging learning environments foster learners’ self-efficacy and encourage IDLE experiences, making a significant contribution to satisfaction and success in online courses.

Furthermore, the findings from this research underscore the significance of IDLE for EFL learners. Educators should actively encourage learners to engage in informal language learning activities through various digital platforms and resources. By promoting IDLE, educators can enhance learners’ language proficiency, autonomy, and sense of belonging in the online language learning community. Given the mediating role of online self-regulated learning, educators should concentrate on teaching self-regulatory skills and strategies to learners. Providing explicit instruction and guidance on goal-setting, time management, and self-monitoring can empower learners to take control of their learning process and optimize their learning outcomes. This approach enhances their satisfaction with online courses and supports their academic success. In addition, policymakers and educational stakeholders can draw upon the insights from this research to develop evidence-based interventions and instructional strategies that support EFL learners in the online language learning context. By investing in research-backed initiatives, institutions can enhance the quality and effectiveness of online language instruction, ultimately leading to higher learner satisfaction and achievement.

## 8 Limitations

However, it is important to acknowledge several limitations in this study. Firstly, the findings presented here are derived from a specific sample of intermediate-level Chinese EFL college students attending various national universities. Although this specificity offers a focused perspective, it raises concerns about the extent to which the results can be generalized to other populations and language learning contexts. Given potential variations in learner profiles, needs, and experiences, it is advisable to exercise caution when extending these findings to diverse learner groups.

Secondly, the cross-sectional nature of this study, while providing a snapshot of relationships between the examined constructs, inherently constrains the establishment of causal links between variables. To gain a more profound understanding of temporal dynamics and causal pathways, future research could benefit from adopting longitudinal research designs. Such an approach could unveil the nuanced cause-and-effect relationships among online learning self-efficacy, IDLE, online self-regulated learning, and course satisfaction. Additionally, another notable limitation lies in the study’s reliance on self-reported data, which introduces the potential for response bias. Although self-reports provide valuable insights into learners’ perceptions, attitudes, and behaviors, they may not always perfectly align with objective measures. To enhance the validity and reliability of findings, future research might consider complementing self-report data with objective assessments and observations.

Building on the insights gained from this study, promising future research directions emerge. Researchers can contemplate intervention studies designed to enhance learners’ self-efficacy, foster more effective IDLE experiences, and promote online self-regulated learning skills. Longitudinal investigations could monitor the long-term impact of such interventions on learners’ satisfaction, academic achievement, and persistence in the online language learning environment. Furthermore, conducting comparative studies across various cultural and language contexts offers valuable insights into the generalizability and applicability of the theoretical relationships explored in this research. These future directions contribute to the expansion of our knowledge and the ongoing improvement of online language learning experiences for diverse learner populations.

## Data availability statement

The original contributions presented in this study are included in this article/Supplementary materials, further inquiries can be directed to the corresponding author.

## Ethics statement

The studies involving humans were approved by the School of Foreign Languages and Literatures, Chongqing Normal University, Daxuecheng, Chongqing 401331, China. The studies were conducted in accordance with the local legislation and institutional requirements. The participants provided their written informed consent to participate in this study.

## Author contributions

YZ: Conceptualization, Data curation, Formal analysis, Investigation, Methodology, Project administration, Resources, Supervision, Visualization, Writing–original draft. AX: Data curation, Formal analysis, Investigation, Methodology, Resources, Software, Validation, Visualization, Writing–review & editing.
