# Supplementary material for: A structural equation model of online learning: investigating self-efficacy, informal digital learning, self-regulated learning, and course satisfaction
Source: Front Psychol. 2024 Jan 11;14:1276266. doi: 10.3389/fpsyg.2023.1276266 (PMC10809156; doi:10.3389/fpsyg.2023.1276266)
Supplement: Supplementary file 1 [file Data_Sheet_1.docx]

**Appendix**

**A: Online Course Satisfaction Scale (OCSS)**

1. I am satisfied with the instructional style.
2. I am satisfied with the learning content and course structure.
3. I am satisfied with the instructors and teaching assistants.
4. I am satisfied with the use of online discussion forum.
5. I am satisfied with the group projects for the course assignment and the criteria of group projects.
6. I am satisfied with the midterm exam and final exam.
7. Overall, I am satisfied with this course.

**B: IDLE Scale**

1. I play games in English.
2. I listen to English language news programmes online or on TV.
3. I listen to songs in English.
4. I listen to English podcasts.
5. I watch English comics online or on TV.
6. I watch sports events in English online or on TV.
7. I watch English language movies or dramas with subtitles in English.
8. I chat with others in English via social media (e.g. Facebook, KaKaoTalk, Line, WeChat, WhatsApp).
9. I Skype with others in English.
10. I send an email to others in English.
11. I share English contents online.
12. I use technology to connect with native speakers of the language (e.g. British, American).
13. I use technology to connect with non-native speakers of English all over the world (e.g. Japanese, Chinese).

**C: Online Self-Regulated Learning Questionnaire (OSLQ)**

1. I set standards for my assignments in online courses.
2. I set short-term (daily or weekly) goals as well as long-term goals (monthly or for the
3. semester).
4. I keep a high standard for my learning in my online courses.
5. I set goals to help me manage studying time for my online courses.
6. I don’t compromise the quality of my work because it is online.
7. I choose the location where I study to avoid too much distraction.
8. I find a comfortable place to study.
9. I know where I can study most efficiently for online courses.
10. I choose a time with few distractions for studying for my online courses.
11. I try to take more thorough notes for my online courses because notes are even more important for learning online than in a regular classroom.
12. I read aloud instructional materials posted online to fight against distractions.
13. I prepare my questions before joining in the chat room and discussion.
14. I work extra problems in my online courses in addition to the assigned ones to master the course content.
15. I allocate extra studying time for my online courses because I know it is time-demanding.
16. I try to schedule the same time every day or every week to study for my online courses, and I
17. observe the schedule.
18. Although we don’t have to attend daily classes, I still try to distribute my studying time evenly across days.
19. I find someone who is knowledgeable in course content so that I can consult with him or her when I need help.
20. I share my problems with my classmates online so we know what we are struggling with and how to solve our problems.
21. If needed, I try to meet my classmates face-to-face.
22. I am persistent in getting help from the instructor through e-mail.
23. I summarize my learning in online courses to examine my understanding of what I have learned.
24. I ask myself a lot of questions about the course material when studying for an online course.
25. I communicate with my classmates to find out how I am doing in my online classes.
26. I communicate with my classmates to find out what I am learning that is different from what they are learning.

**D: Online Learning Self-Efficacy Scale (OLSS)**

1. Willing to face challenges
2. Create a plan to complete the given assignments
3. Willingly adapt my learning styles to meet course expectations
4. Understand complex concepts
5. Keep up with course schedule
6. Evaluate assignments according to the criteria provided by the instructor
7. Complete an online course with a good grade
8. Pay attention to other students’ social actions
9. Initiate social interaction with classmates
10. Apply different social interaction skills depending on situations
11. Develop friendship with my classmates
12. Send email to others with or without attached files
13. Reply to others’ messages in a discussion board
14. Post a new message in a discussion board
15. Clearly ask my questions to instructor
16. Seek help from instructor when needed
17. Timely inform the instructor when unexpected situations arise
18. Initiate discussions with the instructor
19. Express my opinions to instructor respectfully
20. Actively participate in online discussions
21. Effectively communicate with my classmates
22. Respond to other students in a timely manner
23. Request help from others when needed
24. Express my opinions to other students respectfully
25. Provide help to other students when assistance is needed
